# Research on the Human Motion Recognition Method Based on Wearable

**DOI:** 10.3390/bios14070337

**Published:** 2024-07-10

**Authors:** Zhao Wang, Xing Jin, Yixuan Huang, Yawen Wang

**Affiliations:** School of Electrical and Electronic Engineering, Changchun University of Technology, Changchun 130012, China; 2202204104@stu.ccut.edu.cn (Z.W.); 2202204003@stu.ccut.edu.cn (Y.H.); 2202304106@stu.ccut.edu.cn (Y.W.)

**Keywords:** wearable devices, sensors, action recognition, threshold value

## Abstract

The accurate analysis of human dynamic behavior is very important for overcoming the limitations of movement diversity and behavioral adaptability. In this paper, a wearable device-based human dynamic behavior recognition method is proposed. The method collects acceleration and angular velocity data through a six-axis sensor to identify information containing specific behavior characteristics in a time series. A human movement data acquisition platform, the DMP attitude solution algorithm, and the threshold algorithm are used for processing. In this experiment, ten volunteers wore wearable sensors on their bilateral forearms, upper arms, thighs, calves, and waist, and movement data for standing, walking, and jumping were collected in school corridors and laboratory environments to verify the effectiveness of this wearable human movement recognition method. The results show that the recognition accuracy for standing, walking, and jumping reaches 98.33%, 96.67%, and 94.60%, respectively, and the average recognition rate is 96.53%. Compared with similar methods, this method not only improves the recognition accuracy but also simplifies the recognition algorithm and effectively saves computing resources. This research is expected to provide a new perspective for the recognition of human dynamic behavior and promote the wider application of wearable technology in the field of daily living assistance and health management.

## 1. Introduction

With the rapid development and widespread use of the internet, intelligent hardware, and wearable technology, wearable devices have emerged as one of the most promising fields [1]. They are extensively utilized in various sectors, including the medical field [2,3,4,5,6,7,8,9], military applications [10,11,12,13], the sports industry [14,15], film and television production [16,17], and virtual reality environments [18,19]. Some of these products have seamlessly integrated into everyday life, such as smart wristbands [20,21], smart watches [22,23,24], and smart glasses [25,26]. Since the inception of wearable technology by the Massachusetts Institute of Technology in 1960, popular devices such as the Xiaomi smart band, Huawei smart band, and Apple smart band have become prevalent in today’s market. Wearable devices are multifunctional tools that facilitate human–computer interaction, data exchange, and software support to enhance daily living experiences for users with greater convenience and intuitive usability effects [27,28].

Human movement is typically characterized by a single human posture, and a series of postures represent various attitudes and behaviors. Therefore, obtaining data on human movements and postures is highly important [29]. When wearable devices are integrated with human movement, they enable the recognition of human movement, providing valuable information about the physiological and psychological state of individuals. As such, human movement recognition plays a crucial role in daily life [30]. In recent years, numerous researchers both domestically and internationally have focused on developing effective systems for recognizing human movement. Currently, motion state recognition can be broadly categorized into two directions: computer vision-based recognition and sensor-based recognition. For instance, Yadav et al. [31] utilized drone cameras to capture activity videos and employed the sparse weighted temporal attention (SWTA) module along with convolutional neural networks for activity recognition, demonstrating high performance. Similarly, He et al. [32] designed a dual-view adaptive neural network where the view-adaptive model can transform various views into more consistent virtual viewpoints. The experimental results indicate that this framework achieves advanced performance. Furthermore, Gholamiangonabadi et al. [33] utilized a convolutional neural network (CNN) and a signal-personalized human activity recognition (HAR) model to extract features from multimodal sensor data for activity recognition. In signal processing applications, its performance surpasses that of the most advanced CNN method with time-domain characteristics. Additionally, Nafea et al. [34] collected data using accelerometers and gyroscopes to identify daily activities through a novel method employing convolutional neural networks. They captured features at different resolutions using various kernel dimensions as well as two-way long short-term memory (BiLSTM), efficiently selecting the best video while extracting spatial and temporal features, resulting in high accuracy.

As seen from the literature review above, although vision-based recognition algorithms are becoming more mature, their application is limited to specific circumstances and involves personal privacy issues. In contrast, sensor-based approaches are more cost-effective, have easy data collection, are less affected by the environment, and better protect personal privacy. In this field, research has generally focused on the use of a single or small number of sensors to capture motion information. For example, Qu et al. [35] used a single six-axis inertial measurement unit to collect data and analyzed it in combination with an SVM and a quadratic threshold judgment algorithm. Shen et al. [36] used a single three-axis acceleration sensor to monitor lumbar acceleration and adopted threshold classification to identify four activity modes, which also achieved good results. However, these studies that rely on a single sensor have limitations, such as insensitivity to subtle motion capture and limited body part data collection, resulting in poor recognition accuracy of some movements and easy confusion of similar movements. Moreover, recognition algorithms using even a small number of sensors are complex. In response to the above issues, to improve the recognition accuracy and reduce the complexity of the algorithm, this paper designs a wearable human movement recognition method. It obtains data by wearing a wearable transmission device at the corresponding position of the body. A human motion monitoring data acquisition platform is built and composed of a microcontroller circuit, sensor circuit, voltage regulator circuit, charging circuit, and communication circuit. It was connected to the host computer in Type-C mode. Real-time data detection, data fusion and filtering, action judgment by the threshold algorithm, and real-time display by the host computer are completed. The experimental results show that this method has a good recognition effect, reduces the complexity of the algorithm, and can be applied to the fields of daily movement recognition and sports recognition.

## 2. Materials and Methods

### 2.1. Experimental Equipment

**Figure 1:**
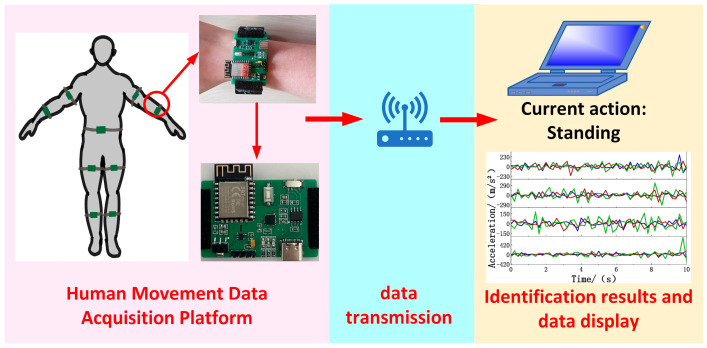
Overall system block diagram.

The wearable human movement recognition method designed in this paper consists of nine wearable devices worn on the left or right upper arm, forearm, thigh, calf, or waist center to collect movement data. When the human movement data acquisition platform completed the data collection, the data were transmitted to a computer, as shown in Figure 1.

#### 2.1.1. Design of the Human Movement Data Acquisition Platform

**Figure 2:**
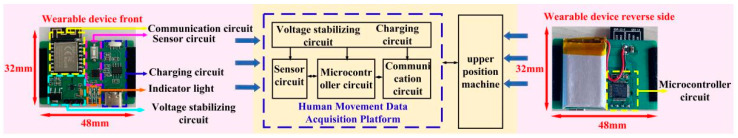
Schematic diagram of the platform hardware.

The human action data acquisition platform is composed of a microcontroller circuit, sensor circuit, voltage regulator circuit, charging circuit, and communication circuit, as shown in Figure 2.

This design uses STM32F103C6T6A (selected from stmicroelectronics, Geneva, Switzerland) as the core processor of the microcontroller circuit. The microcontroller circuit plays a core role in the system, mainly in control, detection, data processing, communication, etc. The device connected to the I/O port of the single-chip microcomputer includes the MPU6050 attitude sensor of the sensor circuit (selected from InvenSense, Sunnyvale, CA, USA), the LED indicator light, and the communication module ESP8266-12 (selected from Lexin Information Technology Co., LTD., Shanghai, China). The microcontroller circuit is visible in the yellow box on the right in Figure 2. The charging circuit is mainly composed of a TP4056 (selected from Topin Microelectronics Co., LTD., Nanjing, China) module powered by 5 V. The Type-C DC input 5 V power supply is connected to the power supply port of the circuit to realize the charging function. The charging circuit is visible in the dark blue box on the left in Figure 2. Since the system’s microcontroller chip, MPU6050 six-axis sensor, and Wi-Fi module require 3.3 V of DC input, the 5 V DC source needs to be stabilized at 3.3 V by a voltage regulator output. The sensor used in the sensor circuit is the MPU6050 six-axis attitude sensor, which is a powerful six-axis inertial measurement unit (IMU). The voltage regulator circuit is visible in the light blue box on the left in Figure 2. The sensor adopts an InvenSense MPU6050 six-axis attitude sensor. In Figure 2, the left pink box graphically marks the position of the MPU6050 in the sensor circuit and its functional role. The data collected by the MPU6050 can realize real-time acquisition of human posture (such as tilt angle and rotation speed), providing a solid data foundation for subsequent motion recognition algorithms, sports health monitoring, and even human–computer interaction design. Finally, the communication circuit exchanges data between the platform and PC software to complete the data transmission. The communication circuit is visible in the left yellow box in Figure 2.

#### 2.1.2. Software Host Computer Design

**Figure 3:**
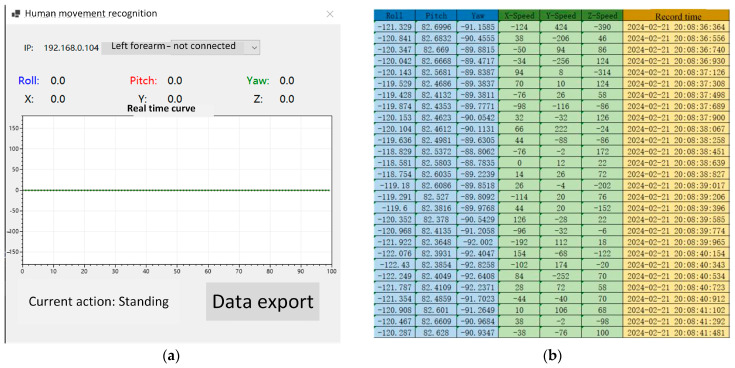
Upper computer software: (**a**) Upper computer interface and (**b**) data storage interface.

The host computer design language is written based on C#, and the host computer design is completed according to the functional requirements of the system, which are divided into five main areas: the data selection area, the data display area, the data mapping area, the identification of the results display area, and the data storage area, as shown in Figure 3a.

- Data selection area: The IP address currently connected to the human motion recognition software is displayed, and the sensor device is connected to the human motion recognition software. The function of data selection can be achieved using the lower pull bar, which can select nine different body parts.
- Data exhibition area: The acceleration data and angular velocity data of the current human movement are displayed in the form of two rows and three columns to update the data movement information of the current user in real time.
- Data display area: The acceleration data of the data display area are displayed in the form of data lines.
- Recognition result display area: When the human body performs different actions, the action recognition result will be displayed in this area.
- Data storage area: The movement data under different movements of the human body can be stored. Different movement data of different body parts can be selected according to the data selection area. After freely setting the storage path, the acceleration and angular velocity data under the current motion state are stored as a table, as shown in Figure 3b.

### 2.2. Human Motion Recognition Algorithm

#### 2.2.1. DMP Attitude Solution Algorithm

In this paper, the DMP attitude solution algorithm is used to determine the attitude of the MPU6050 six-axis sensor, which is mainly divided into the data acquisition stage, the quaternion integration stage, the accelerometer calibration stage, and the filtering stage.

1. Data Acquisition phase

In the data acquisition phase of the DMP algorithm, the angular velocity and linear acceleration of the six-axis sensor become the basis of the attitude calculation. The course angle yaw(y) is assumed to rotate around the Z-axis; the pitch angle (p) rotates around the Y-axis. Rotation around the *X*-axis is called the roll angle (r), and the three-axis gyroscope provides information about the device’s rotation speed by measuring the device’s angular speed. Its output vector is $\left[\begin{array}{ccc} \omega_{x} & \omega_{y} & \omega_{z} \end{array}\right]$.

Moreover, the three-axis accelerometer provides information related to the device’s orientation and direction of motion by measuring the device’s linear acceleration in space. The output vector of the accelerometer is $\left[\begin{array}{ccc} a_{x} & a_{y} & a_{z} \end{array}\right]$, representing the linear acceleration of the device on the three coordinate axes. This dataset is a critical input in attitude calculations, especially in situations involving changes in device acceleration.

2. Quaternion integration stage

One of the cores of the DMP algorithm is quaternion integration, which updates the rotation state of the device by using the angular velocity information provided by the gyroscope. A quaternion is a mathematical tool used to represent rotation, which can effectively avoid the singularity problem in the attitude solution and improve the stability of the solution. The differential equation for the quaternion integral is as follows:

(1) $$\frac{dq}{dt}=\frac{1}{2}\Omega (q)\omega$$

Here, q = $\left[\begin{array}{cccc} q_{0} & q_{1} & q_{2} & q_{3} \end{array}\right]$ represents the quaternion, Ω(q) is the rotation matrix of the quaternion, and ω is the angular velocity. Through the numerical integration method, it is possible to calculate the change in quaternion under a discrete time step to obtain the device’s rotation state in space. In this stage, the algorithm constantly updates the quaternion, simulates the device’s rotation, and ensures the accuracy and real-time performance of the solution. This step is the basis of the attitude calculation and provides accurate initial data for subsequent calibration and filtering.

3. Accelerometer calibration phase

Accelerometer calibration is performed to eliminate errors due to gravity and nonideal motion of the equipment. Through the linear acceleration data provided by the accelerometer, the DMP algorithm calibrates the quaternion, corrects the influence of the accelerometer, and ensures the accuracy of the attitude solution. The output vector of an accelerometer $\left[\begin{array}{ccc} a_{x} & a_{y} & a_{z} \end{array}\right]$ should be affected only by gravity at rest, while it may be subject to additional acceleration in a dynamic environment.

First, the value collected by the three-axis accelerometer is converted into a unit vector.

(2) $$a_{x}=\frac{a_{x}}{\sqrt{{{(a}_{x})}^{2}{+(a}_{y}{)}^{2}{+(a}_{z}{)}^{2}}}$$

(3) $$a_{y}=\frac{a_{y}}{\sqrt{{{(a}_{x})}^{2}{+(a}_{y}{)}^{2}{+(a}_{z}{)}^{2}}}$$

(4) $$a_{z}=\frac{a_{z}}{\sqrt{{{(a}_{x})}^{2}{+(a}_{y}{)}^{2}{+(a}_{z}{)}^{2}}}$$

The gravity vector derived from the integrated attitude of the gyroscope is

V_x_ = 2(q_1_q_3_ − q_0_q_2_) (5)

V_y_ = 2(q_0_q_1_ + q_2_q_3_) (6)

(7) $$V_{z}{=q}_{0}^{2}{-q}_{1}^{2}{-q}_{2}^{2}{+q}_{3}^{2}$$

a_x_, a_y_, and a_z_ are gravity vectors measured by the accelerometer on the coordinate reference frame, which is the actual measured gravity vector. V_x_, V_y_, and V_z_ are gravity vectors derived from the integrated attitude of the gyroscope, and they are all gravity vectors on the frame of reference of the body coordinates. Then, an error vector can be defined

(8) $$e_{x}=(a_{y}v_{z}-a_{z}v_{y})$$

(9) $$e_{y}=(a_{z}v_{x}-a_{x}v_{z})$$

(10) $$e_{z}=(a_{x}v_{y}-a_{y}v_{x})$$

e_x_, e_y_, and e_z_ represent the cross-products of two gravitational vectors. This cross-product vector is still located in the body coordinate system, the gyro integration error is also in the coordinate system, and the size of the cross-product is proportional to the gyro integration error, which can be used to correct the gyroscope.

The integral error proportional integral gain is defined as follows:

e_x_Int = e_x_Int + e_x_∗K_i_ (11)

e_y_Int = e_y_Int + e_y_∗K_i_ (12)

e_z_Int = e_z_Int + e_z_∗K_i_ (13)

where K_i_ is the convergence of the gyroscope bias of the integral gain domination rate.

Then, for the modified gyroscope:

g_x_ = g_x_ + K_p_∗e_x_ + e_x_Int (14)

g_y_ = g_y_ + K_p_∗e_y_ + e_y_Int (15)

g_z_ = g_z_ + K_p_∗e_z_ + e_z_Int (16)

where K_p_ is the proportional gain domination rate converging to the accelerometer and g_x_, g_y_, and g_z_ are the adjusted gyroscope measurements.

The quaternion differential equations are solved using the first-order Rungokuta method:

(17) $$q_{0}=q_{0}+(-q_{1}*g_{x}-q_{2}*g_{y}-q_{3}*g_{z})*\mathrm{halfT}$$

(18) $$q_{1}=q_{1}+(q_{0}*g_{x}+q_{2}*g_{z}-q_{3}*g_{y})*\mathrm{halfT}$$

(19) $$q_{2}=q_{2}+(q_{0}*g_{y}-q_{1}*g_{z}+q_{3}*g_{x})*\mathrm{halfT}$$

(20) $$q_{3}=q_{3}+(q_{0}*g_{z}+q_{1}*g_{y}-q_{2}*g_{x})*\mathrm{halfT}$$

where halfT is half of the sampling period.

Then, it is converted to the Euler angle by the rotation matrix

(21) $$y=\arctan\frac{2(q_{0}q_{3}+q_{1}q_{2})}{q_{0}^{2}{+q}_{1}^{2}-q_{2}^{2}{-q}_{3}^{2}}$$

p = arcsin2(q_0_q_2_ – q_1_q_3_) (22)

(23) $$r=\arctan\frac{{2(q}_{0}q_{1}{+q}_{2}q_{3})}{q_{0}^{2}{-q}_{1}^{2}{-q}_{2}^{2}{+q}_{3}^{2}}$$

The calibration process mainly identifies and eliminates the gravity component by analyzing the output of the accelerometer in the static state to obtain the actual linear acceleration. Adjusting the quaternions to be consistent with the calibrated accelerometer data achieves a more accurate estimation of the device’s attitude.

4. Filtering phase

In the filtering stage, the goal is to smooth the fused data by using a filtering algorithm to reduce noise and error and improve the accuracy and stability of the attitude estimation.

In the filtering process, the DMP algorithm, called the internal Kalman filtering algorithm, comprehensively considers sensor noise, the system model, and prior information and effectively filters out unnecessary interference through state estimation updates. At the same time, their parameters are adjusted according to the actual situation for flexible optimization. This enables the algorithm to adapt to various environments and application scenarios, ensuring accurate and stable attitude information in dynamic environments.

In the Kalman filtering algorithm, the main filtering process is as follows:

First, its equation of state is:

X_k_ = A_k_∗X_k−1_ + B_k_∗U_k−1_ + W_k−1_ (24)

X_k_ is the state vector of the system, representing the state of the system at time step k. A_k_ is a state transition matrix that describes the state transition of the system from time step K − 1 to k. B_k_ is the control input matrix, representing the influence of external inputs on the system’s state. U_k−1_ is the control input vector. W_k−1_ is the system process noise, representing the uncertainty of the system state.

Its prediction equation is:

Z_k_ = H_k_∗X_k_ + V_k_ (25)

Z_k_ is the observed value measured at time step k (sensor measurement). H_k_ is an observation matrix that describes the relationship between the state of the system and the observations. V_k_ is the observation noise, representing the observed value’s uncertainty.

The main steps of Kalman filtering can be divided into prediction and update steps.

In the prediction step

(26) $$X_{\bar{k}}=A_{k}*X_{k-1}+B_{k}*U_{k-1}$$

(27) $$P_{\bar{k}}=A_{k}*P_{k-1}*A_{k}^{T}+Q_{k}$$

$X_{\bar{k}}$ is the prior estimate (predicted value) of the state of time step k. $P_{\bar{k}}$ is a prior estimated covariance matrix for the state of time step k. $Q_{k}$ is the covariance matrix of the system process noise.

In the update step

(28) $$K_{k}=P_{\bar{k}}*H_{k}^{T}*{\left(H_{k}*P_{\bar{k}}*H_{k}^{T}+R_{k}\right)}^{-1}$$

(29) $$X_{k}=X_{\bar{k}}+K_{k}*(Z_{k}-H_{k}*X_{\bar{k}})$$

(30) $$P_{k}=(I-K_{k}*H_{k})*P_{\bar{k}}$$

$K_{k}$ is the Kalman gain of time and step k. $R_{k}$ is the covariance matrix of the observed noise. $X_{k}$ is a posterior estimate (update value) of the state of time step k. I is the identity matrix. $P_{k}$ is the posterior estimation covariance matrix of the state of time step k.

#### 2.2.2. Determination of the Motion State of the Threshold Algorithm

The system collects data from the sensors in each part of the human body and determines the acceleration and angle information according to the feedback from each sensor. The upper computer collects and processes this part for each data part. First, the data are usually received when the upper computer is connected to the nine parts of the human body. The determination of human posture mainly relies on the acceleration data of nine sensors to determine the standing, walking, and jumping states. In the standing state, the body sensor data are relatively stable; when the human body is walking, the left and right thighs, calves, and lower arms are almost in motion, and the acceleration data will show a certain amplitude of change. When the human body jumps, the number of sensor movements increases. In summary, the current motion state of the human body can be determined according to the number of sensors in motion. The threshold algorithm is combined with the sensor motion data with the following algorithms:

1. Input data

The x-, y-, and *z*-axis accelerations a_1_, a_2_, and a_3_ of a specific body part at a specific time are obtained by taking the acceleration data of different body parts in human movement at a specific time after the above data fusion and filtering processing. The absolute values of a_1_, a_2_, and a_3_ are calculated and processed to obtain V_1_, V_2_, and V_3_.

2. Threshold setting

In setting a threshold, the recognition effect is tested by setting different threshold values. Assume that the threshold is A.

- When the value set by the threshold is greater than A, due to the high threshold value, only the jumping action can exceed the threshold to carry out the internal cycle of the algorithm. In contrast, the number of walking and standing actions is mostly less than the set threshold, resulting in poor recognition of walking and standing actions.
- When the threshold is less than A, because the threshold is too low, large numbers of walking and jumping actions can exceed the set threshold during the algorithm, resulting in confusion between walking and jumping actions and a poor recognition effect, and only standing actions have a good recognition effect.
- When the threshold value is set to A, this method has an ideal recognition effect on standing, walking, and jumping actions. In summary, when the test threshold is A, the recognition effect of this method is relatively ideal.

3. Output identification results

V_1_, V_2_, and V_3_ are compared with threshold A to obtain the human movement at a certain time. When any value of a_1_, a_2_, and a_3_ is greater than A, the number of A wearable devices i is recorded, and the number of i is accumulated in a cycle until any value of a_1_, a_2_, or a_3_ is less than a, and the cycle ends.

**Figure 4:**
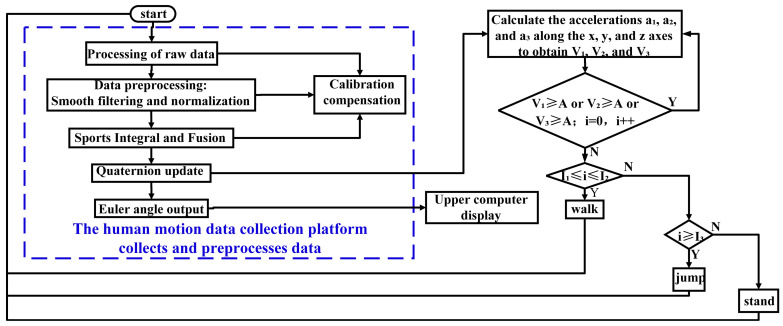
Algorithm flow chart.

- When I_1_ ≤ i ≤ I_2_, the output body is currently walking.
- When i ≥ I_3_ is used, the current output of the human body is the jumping action; otherwise, the current output of the human body is the standing action. The algorithm flow chart is shown in Figure 4 (I_1_, I_2_, and I_3_ are the set numbers of sensors).

## 3. Results

### 3.1. Experimental Design

This experiment collected exercise data from 10 volunteers (5 males and 5 females, aged 23~30 years, with heights of 160~183 cm, and in good physical condition) in the corridors and laboratories of the school. The volunteers wore the wearable device on the corresponding fixed parts of the body and collected data on standing, walking, and jumping movements. Each volunteer repeated the above three movements 20 times each. A total of 600 motion classification data points were collected.

### 3.2. Experimental Results and Analysis

**Figure 5:**
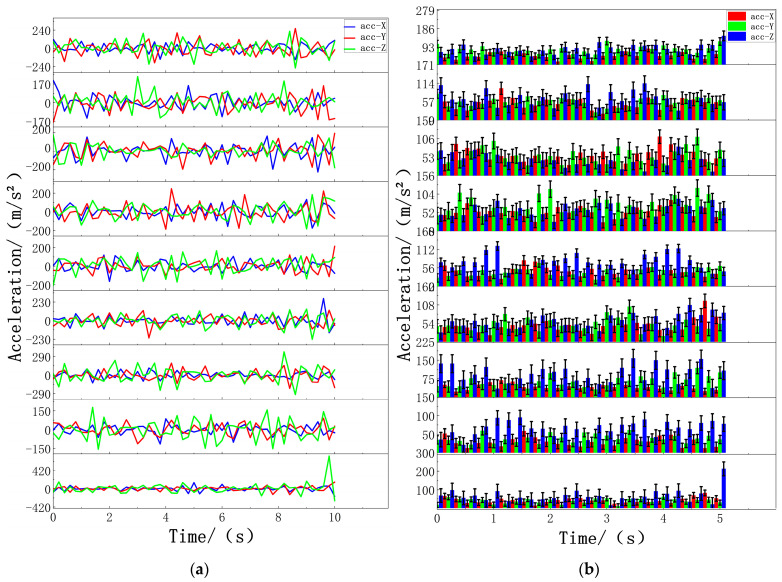
Standing data of nine sensors: (**a**) acceleration standing data of nine sensors and (**b**) standing data on the absolute acceleration values of nine sensors. The nine figures from top to bottom are data graphs of the following parts: left forearm, left upper arm, right forearm, right upper arm, middle waist, left calf, left thigh, right calf, and right thigh.

**Figure 6:**
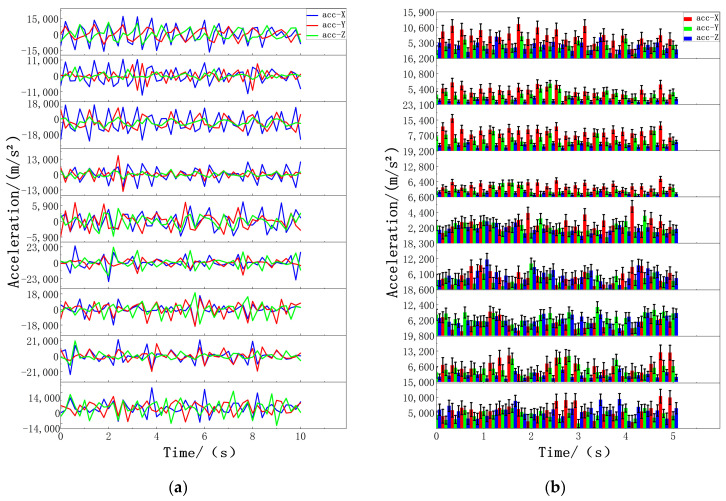
Walking data of nine sensors: (**a**) Acceleration walking data of nine sensors. (**b**) Absolute acceleration walking data of nine sensors. The nine figures from top to bottom are data graphs of the following parts: left forearm, left upper arm, right forearm, right upper arm, middle waist, left calf, left thigh, right calf, and right thigh.

**Figure 7:**
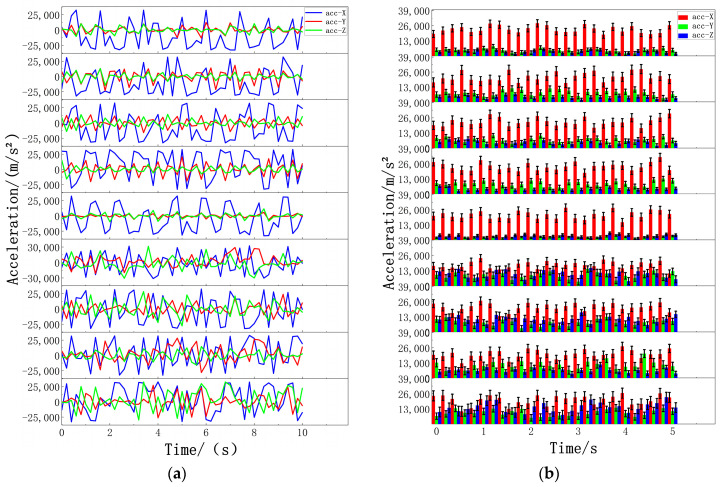
Jump data of nine sensors: (**a**) Acceleration jump data of nine sensors. (**b**) Jumping data of absolute acceleration values from nine sensors. The nine figures from top to bottom are data graphs of the following parts: left forearm, left upper arm, right forearm, right upper arm, middle waist, left calf, left thigh, right calf, and right thigh.

In this experiment, after volunteers wore wearable devices on the corresponding parts of the body, the data on volunteers’ standing, walking, and jumping movements were collected at different times and places. The threshold algorithm was run on the computer terminal of the Microsoft Windows 10 operating system, and the three kinds of motor action classifications of volunteers were tested through the fusion of the threshold algorithm and data filtering. This experiment takes 0.2 s as the time interval for data collection. The collected motion data are as follows. In the standing state, the acceleration data of each part change gradually, and the values after absolute value processing are concentrated in the range of 20 m/s^2^ to 200 m/s^2^. Most sensor data also reflect this trend, as shown in Figure 5. During walking, the acceleration of specific body parts fluctuates, and after absolute value processing, the value range expands from 2000 m/s^2^ to 10,000 m/s^2^. The data of different sensors at the same time point were distributed in two ranges, from 2000 m/s^2^ to 5000 m/s^2^ and from 5000 m/s^2^ to 10,000 m/s^2^, as shown in Figure 6. For jumping movements, the acceleration data of almost all body parts showed significant fluctuations, the absolute value increased significantly to between 9000 m/s^2^ and 25,000 m/s^2^, and most sensors recorded values exceeding 10,000 m/s^2^, as shown in Figure 7.

Six hundred recognition experiments were conducted for each of the standing movements, walking movements, and jumping movements, and 1800 recognition experiments were conducted for the three movements. When the recognition results are consistent with the performance of the action currently in progress, we consider the recognition to be correct. The percentage of the number of experiments with correct identification to the total number of experiments for each action is calculated, that is, the recognition accuracy rate. The statistical data of the test classification results are shown in the table. It can be seen from the table that the recognition accuracies of standing movements, walking movements, and jumping movements are 98.33%, 96.67%, and 94.60%, respectively, with an average recognition rate of 96.53%.

In addition, Table 1 reveals the pattern of misrecognition: standing is often mistaken for walking or jumping, walking may be mistaken for standing or jumping, and jumping can be confused with standing or walking. The misunderstanding between walking and jumping is particularly prominent; that is, walking is misjudged as standing or jumping, and jumping is mistakenly classified as walking frequently. Some of these identification errors are rooted in differences in individual movement habits, speed, and frequency; for example, a small number of people may swing their arms wildly while walking, hold their arms stationary close to their bodies, or even jump forward, which causes diversity in sensor data, occasionally causing misjudgments and confusing the boundaries of various movements. Although misclassification is a small probability event, it will have a ripple effect on practical application: if the athlete’s movement is not standard, the training plan designed by the coach may deviate from the correct direction. In the health management scenario, atypical movements may be misidentified, resulting in inaccurate health assessments after exercise.

**Figure 8:**
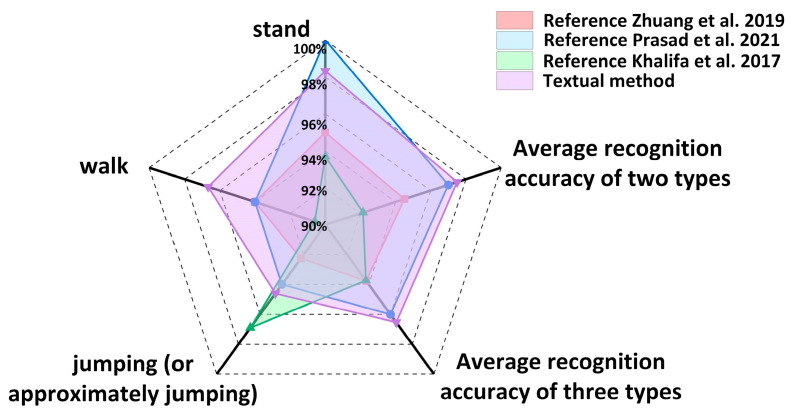
The proposed scheme is compared with other schemes for human motion recognition, [37,38,39].

This paper compares and analyzes relevant literature in the field of human motion recognition. Zhuang et al. [37] used a single six-axis sensor to collect data, combined time domain and frequency domain analysis, and used the multiclass classification technology of a support vector machine for motion pattern recognition. Prasad et al. [38] used smartphone accelerometers to collect data and applied convolutional neural networks for recognition. Khalifa et al. [39] also used mobile phone accelerometers to distinguish different behaviors by setting different thresholds. In this study, studies using different sensor technologies and recognition algorithms were selected to compare the recognition accuracy of standing, walking, and jumping (or approximately jumping) movements, as well as the average recognition accuracy of two or three types of movements. The specific results are shown in Table 2 and Figure 8. The recognition accuracy of [37] is relatively low, and the accuracy of [38] is relatively high, but the algorithm has a certain complexity, and the accuracy of [39] is low during the recognition walk. The results of the data analysis show that the recognition method proposed in this paper is superior to other schemes in terms of recognition efficiency because it not only improves the recognition accuracy but also successfully simplifies the algorithm structure and reduces the need for computing resources.

## 4. Discussion

To improve the recognition accuracy and simplify the algorithm complexity, this paper developed a set of human action recognition systems based on wearable devices, which achieved remarkable results and effectively reduced the computational burden. It is worth noting that the current research is limited to motion recognition in the laboratory environment; although the sensor shows strong environmental adaptability, its universality needs to be validated in outdoor or variable scenarios. Future research is expected to expand to experiments in a variety of environments, including more types of motion for identification testing, to explore and optimize the overall performance of the system.

## 5. Conclusions

In summary, this paper proposes an innovative wearable human movement recognition strategy that focuses on the accurate recognition of three basic movements: standing, walking, and jumping. With the help of a human movement data acquisition system to complete the data collection, the DMP platform was used for data purification and integration, and the threshold algorithm was adopted to implement the action classification. The experimental results show that the recognition accuracy of the strategy for three kinds of actions reaches 98.33%, 96.67%, and 94.60%, and the comprehensive recognition accuracy reaches 96.53%. The comparison with other existing recognition schemes further confirms the superiority of this method, especially in distinguishing the three motion states. This wearable motion recognition technology has a wide range of practical applications and can not only meet the needs of personalized customization but also achieve universal deployment, providing more personalized and efficient daily assistance and health management solutions for users.

## Figures and Tables

**Table 1:** Classification results of human motion state recognition.

Test Motor Action (Unit: Unit)		Identification Result			Recognition Rate (%)
		Stand (Unit: Unit)	Walk (Unit: Unit)	Jump (Unit: Unit)	
Stand	600	590	8	2	98.33%
Walk	600	17	580	3	96.67%
Jump	600	6	26	568	94.60%

**Table 2:** Comparison of literature recognition accuracy.

	Reference [37]	Reference [38]	Reference [39]	Textual Method
Stand	95.00%	100.00%	93.70%	98.33%
Walk	94.00%	94.00%	90.60%	96.67%
jumping (or approximately jumping)	92.25%	94.00%	96.90%	94.60%
Average recognition accuracy of two types	94.50%	97.00%	92.15%	97.50%
Average recognition accuracy of three types	93.75%	96.00%	93.73%	96.53%

## Data Availability

The data that support the findings of this study are available from the corresponding authors upon reasonable request.
